# The effect of a mindfulness-based therapy on different biomarkers among patients with inflammatory bowel disease: a randomised controlled trial

**DOI:** 10.1038/s41598-020-63168-4

**Published:** 2020-04-08

**Authors:** Rafael González-Moret, Ausias Cebolla, Xavier Cortés, Rosa M. Baños, Jaime Navarrete, José Enrique de la Rubia, Juan Francisco Lisón, José Miguel Soria

**Affiliations:** 1 Department of Biomedical Sciences, Universidad Cardenal Herrera-CEU Universities, Valencia, Spain; 2 Institute of Biomedical Sciences, Universidad Cardenal Herrera-CEU Universities, Valencia, Spain; 3 Odisesas Institute, Universidad CEU Cardenal Herrera-CEU Universities, Valencia, Spain; 4 Department of Nursing, Universidad Cardenal Herrera-CEU, CEU Universities, Castellon, Spain; 5 Internal Medicine Service, Digestive Medicine Section, Hospital Universitario de Sagunto, Valencia, Spain; 6 Department of Medicine, Universidad Cardenal Herrera-CEU, CEU Universities, Valencia, Spain; 7 Department of Personality, Evaluation, and psychological treatments, Universidad de Valencia, Valencia, Spain; 8 Department of Nursing, Catholic University San Vicente Martir, Valencia, Spain; 9 Obesity and Nutrition Pathophysiology CIBER (CB06/03), Instituto Carlos III, Madrid, Spain

**Keywords:** Predictive markers, Prognostic markers, Crohn's disease, Ulcerative colitis

## Abstract

Mindfulness-based interventions have shown some efficacy in decreasing stress levels and improving quality of life. However, so far, only a few studies have studied this type of intervention among patients with inflammatory bowel disease and none of them have studied their effects on inflammatory biomarkers. This current study was a two-armed, single-centre, randomised (2:1 ratio) controlled trial used to evaluate the effects of a mindfulness-based intervention (*n* = 37) compared to standard medical therapy (*n* = 20) in patients with Crohn’s disease or ulcerative colitis. The mindfulness intervention blended four internet-based therapy modules with four face-to-face support sessions. The outcomes we assessed were faecal calprotectin (primary outcome), C-reactive protein, and cortisol levels measured in hair samples at several timepoints. The between-group analysis highlighted significant decreases in faecal calprotectin and in C-reactive protein levels in the mindfulness-based intervention group compared to the standard medical therapy group at the six-month follow-up (faecal calprotectin: −367, [95% CI: −705, −29], *P* = 0.03; C-reactive protein: −2.82, [95% CI: −5.70, 0.08], *P* = 0.05), with moderate to large effect sizes (faecal calprotectin: ηp^2^ = 0.085; C-reactive protein: ηp^2^ = 0.066). We concluded that mindfulness-based therapy administered as part of standard clinical practice effectively improves inflammatory biomarkers in patients diagnosed with inflammatory bowel disease.

## Introduction

The main feature of Crohn’s disease (CD), inflammatory bowel diseases (IBDs), and ulcerative colitis (UC) is mucosal inflammation which flares-up and abates over time. Researchers in this field have more recently come to appreciate that the outcomes of these diseases can often be improved by targeting treatments towards the bowel inflammation present in these patients, rather than treating only the symptoms of these diseases. This reframing of treatments so that the therapies administered focus on, and are guided by, patient mucosal inflammation has resulted in a resurgence of interest in inflammation biomarkers^1,2^.

Inflammation biomarkers have been widely described as potentially useful in several different clinical scenarios. In the broadest sense, they are usually used for two main reasons: firstly, to identify which patients from among those with symptoms compatible with of IBD should be submitted for further analyses with a view to providing a definitive diagnosis of this disease; secondly, they may be used in patients diagnosed with IBD to measure and/or monitor how the disease activity responds to induction or maintenance therapy. The latter encompasses a wide range of situations which could include the identification of patients who may be starting to relapse while receiving an already-established therapy or whose response to a new treatment is good; checking for potential relapses in patients with CD after surgical resection, and increasingly, screening for any patients with a high probability of suffering a clinical relapse when a given therapy is withdrawn. Of note, different cut-off thresholds are often used in different clinical circumstances to optimise the performance of identical biomarker assays^3–9^.

Patients with IBS frequently live with symptoms of psychological distress, including anxiety or depression and report that their quality of life (QoL) becomes poorer as the severity of their disease increases^10–17^. Moreover there is increasing evidence that experiencing such distress in a continued way could cause IBD activity to increase^18^. Thus, the way that these patients manage stress on a daily basis and during specific life events can aggravate their physical symptoms of the disease and increase their probability of developing comorbidities. Furthermore, inflammatory biomarkers are also altered in people with symptoms of anxiety and depression^19^. In fact, some symptoms associated with mental disorders which are also present in IBD, such as anhedonia, loss of appetite, aching joints, fever, tiredness, exhaustion, and withdrawal from social activities^20^, can be predicted by the levels of these inflammatory markers.

Psychological interventions have been shown to effectively reduce the response of inflammatory biomarkers; indeed, one recent meta-analysis found this effect with a small to medium effect-size in different disorders^21^. However, so far, no studies have analysed the efficacy of the psychological interventions for IBD that were included in this meta-analysis. Nevertheless, mindfulness-based interventions (MBIs), understood as an awareness of the experience of the present moment and emphasising the attention paid to one’s thoughts, bodily sensations, and emotions^22^, have shown to be effective among patients with IBD^18^, with their potential mechanisms described elsewhere^23^. MBIs usually combine meditation with contemporary cognitive-behavioural approaches, and several mechanisms behind their effectiveness have been identified^24,25^. In fact, their positive effects on different mental health conditions have been reported in diverse clinical and non-clinical populations^26^.

Even though MBI represents one the most promising psychological interventions available for IBD, only a few studies have tested the efficacy of these interventions to reduce stress levels and improve QoL in IBD, and none of these included biomarkers among their outcome indicators^27–29^. Thus, in order to better understand the health benefits associated with MBI, it is important to investigate the links between interventions and the responding biological pathways, including inflammatory responses^30^. Therefore, in this study we assessed the effectiveness of a blended MBI intervention and examined its effect on mucosal inflammation by testing inflammation biomarker concentrations in patients diagnosed with IBD. We hypothesised that the blended MBI intervention plus standard medical therapy (SMT) would decrease inflammation biomarker [faecal calprotectin (FC), C-reactive protein (CRP), and cortisol in hair] concentrations compared to SMT alone.

## Methods

### Study design

This two-armed, single centre randomised controlled trial (NCT02963246, 15/11/2016) was approved by the Human Ethics Committee at the Hospital Universitario de Sagunto (Spain) and complied with the ethical guidelines set out in the Declaration of Helsinki. The trial was conducted in the IBD Unit at the Hospital Universitario de Sagunto from May 2017 to March 2018.

Eligible participants were patients aged between 18 and 55 years, diagnosed with CD or UC according to the European Crohn’s and Colitis Organisation (ECCO) criteria, and in clinical remission for the prior 3 months. We also applied the following inclusion criteria: the absence of cognitive impairments; patients with UC with a partial Mayo Score of ≤2 points and with no item scores >1, or patients with CD with a Harvey–Bradshaw score <5 points; experience of at least one inflammatory-activity flare-up in the 12 months prior; no changes in medication types or modifications to their usual treatment in the 3 months prior. Patients with no internet or smartphone access, difficulty in understanding the Spanish language; psychiatric disorders (psychosis, bipolar disease, substance abuse, etc.); suffering a recent emotional shock (e.g. death of a relative or an accident); and individuals who were pregnant at the time of recruitment were excluded from the study. All the study participants signed an informed consent statement before the study commenced.

Before the trial started, a researcher not involved in patient recruitment or inclusion in the study generated a random sequence using a computerised random number generator; this was concealed from all the personnel involved in the study throughout its duration. Upon enrolment in the study, 57 participants were randomly assigned, in a ratio of 2:1, either to the MBI (*n* = 37) or standard medical therapy (SMT; *n* = 20) groups.

The MBI intervention was a blended face-to-face and internet-based intervention comprising four internet-based therapy modules and four in-person support sessions. The face-to-face sessions lasted two hours and were administered at the end of weeks 1, 3, 6, and 8 by a psychologist (A.C.) specialised in group-format MBI; the participants were specifically advised to perform at least three face-to-face sessions (minimum attendance rate). The internet-based sessions were delivered over eight weeks on a one-to-one basis via the internet; the minimum criterion for program completion was participation in a total of four modules (minimum attendance rate). Participants in the MBI group also received SMT; detailed information about the blended intervention described elsewhere^31^.

In the face-to-face sessions the therapist explained the theoretical framework of mindfulness training, mindfulness exercises (including mindful breathing, body scanning, etc.), and the management of the internet-based sessions. The structure of the face-to-face support sessions was: (1) guided meditation; (2) an inquiry process; (3) an analysis of the week’s difficulties; and (4) an explanation of the online tasks to be completed. The final session reviewed what had been learned during the course and prepared the participants to practice these skills outside the group setting. The facilitator was an experienced mindfulness teacher and researcher and had previously taught several instructor courses in different protocols including mindfulness-based stress reduction, mindfulness-based cognitive therapy, cognitive-behavioural conjoint therapy, and contemplative practice-based wellbeing training^32^.

The internet-based one-to-one program was carried out via a web platform (https://www.psicologiaytecnologia.com/) which was accessed using a login and password provided to each of the participants. The platform provided the participants with four different modules: M1. Getting to know mindfulness (week 1); M2. Establishing formal and informal practices for mindfulness (week 2); M3. Thought management, body-scanning practices, and mindfulness values (weeks 3–4); M4. Self-compassion: integrating mindfulness into everyday life (weeks 5–8)^33^. The modules included pre-recorded video and audio meditations and each module lasted approximately 60–90 minutes, depending on the participant’s pace and time availability. All the measurements in this study were assessed before the treatment at baseline and at a 6-month follow-up by researchers who were blinded to the group allocation.

### Outcome measures

The primary outcome of this study was the level of FC, as determined by fluorescent enzyme immunoassay analysis (range: 0–100 mg/g dry weight) using a UniCap 100 analyzer. The secondary outcomes were plasma CRP levels and cortisol levels measured in patient hair samples. CRP concentrations were determined by immunoturbidimetric analysis using a Cobas 8000 analyzer (module c701). In this assay, the reaction of CRP with a specific antibody produces insoluble immune complexes in proportion to the CRP concentration, and these changes in turbidity can be measured spectrophotometrically in milligrams per millilitre. Cortisol levels were measured using a high-sensitivity saliva and hair cortisol enzyme-linked immunosorbent assay (ELISA, Salivary Cortisol ELISA DRG. DRG Instruments GmbH. REF: SLV 2930. LOT: 63K047) kit with a sensitivity of 0.024 ng/ml and a detection range of 0–30 ng/ml, according to the manufacturer’s instructions (DRG Instruments GmbH, Germany).

All of the samples were simultaneously analysed at the end of the study in order to avoid bias derived from using different kit lots^34^. Hair samples were cut from the posterior vertex area of each patient and were individually washed with 2.5 ml of isopropanol with agitation (1800 rpm for 2.5 min) to remove dirt and any external steroids without affecting internal steroids. The samples were dried for 36 h at room temperature and then cut into fragments <2 mm with scissors; 120–150 mg per sample was introduced into microtubes and 1.5 ml of methanol was added, followed by incubation for 18 h at 30 °C with stirring (100 rpm, Mixer block^®^). After centrifuging the vials at 7000 g for 2 min, 750 μl of the liquid phase was recovered and incubated in fresh microtubes at 38 °C until completely dry, and the residue obtained was reconstituted in 0.2 ml of 0.05 M phosphate-buffered saline.

### Sample size

An a priori analysis of the effect size and sample size was conducted applying an α-level of 0.05 for the desired power of 90%, according to the FC levels we saw in a previous pilot study (partial eta-squared [ηp^2^] = 0.058)^35^. Thus, the recruitment target was 23 participants per group (G*Power = 3.0.10); we increased the sample size by 25% to compensate for potential alterations in the statistical significance of the results caused by possible dropouts in the intervention groups, and thus, the final sample size was a total of 57 participants.

### Statistical analysis

Two-way mixed ANCOVA test were used to compare the study effects on FC, CRP, and cortisol levels, using time as the within-group factor (baseline versus follow-up at 6 months), and the group as the between-group factor (MBI versus SMT). The analysis was adjusted for pre-intervention (baseline) data. The effect sizes were estimated using the ηp^2^ and were interpreted following Cohen’s guidelines for small, moderate, and large effect sizes (ηp^2^ = 0.01, 0.06, or 0.14, respectively)^36^. The statistical analyses were performed according to the intention-to-treat analysis using SPSS software (v.22.0) for Windows (SPSS Inc., Chicago, Ill, USA). The data are presented as the mean plus or minus the standard deviation (*SD*), considering the probability of statistical significance (*p*-values) at threshold values of 0.05 or less for all the comparisons.

## Results

**Figure 1:**
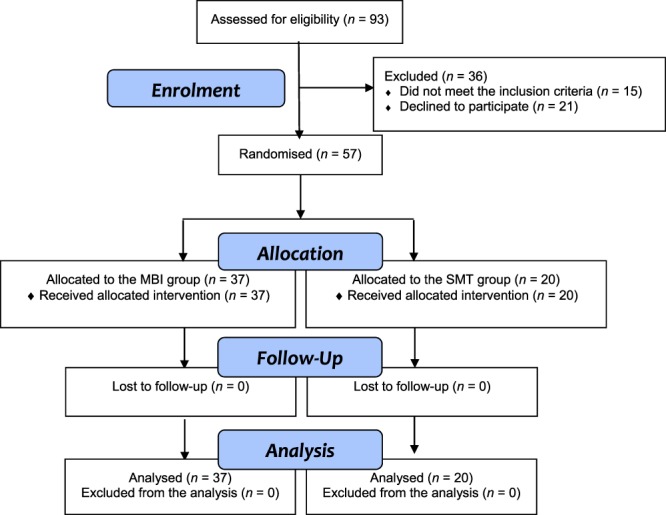
Flow of participants through the trial.

A total of 93 patients were screened for this study; 36 were excluded because they declined to participate (21 patients) or did not meet the inclusion criteria because they had no internet or smartphone access (4); had a psychiatric disorder (4); had suffered a recent emotional shock (2); or their medication had changed in the prior three months (5). Figure 1 details the participant selection process, and Table 1 shows the general characteristics of the study population. A total of 35 out of the 37 patients (95%) attended three or more face-to-face sessions, and 100% of the participants completed all the internet-based modules. The number of flare-ups during the intervention was similar in both groups (3 out of 20 in the SMT group vs. 5 out of 37 in the MBI group). Three patients (2 in IBD and 1 in SMT) decreased their medical treatment during the study period. The between-group analysis highlighted significant decreases in FC and CRP levels in the MBI group compared to the SMT group at 6 months’ follow-up, with moderate to large effect sizes (Table 2). In addition, there was a slight decrease in the hair cortisol levels in the MBI group compared to the SMT group (with a small to moderate effect size) at 6 months, but this difference was not significant.

**Table 1:** Baseline clinical characteristics of the study participants.

VARIABLES	GROUP	
	MBI (*n* = 37)	SMT (*n* = 20)
Age (year)	46.2 (10.9)	46.3 (11.9)
Sex (men/women)	8/29	11/9
Faecal calprotectin (µg/g)	198 (394)	222 (242)
C-reactive protein (mg/dL)	1.93 (2.50)	2.46 (3.81)
Cortisol in hair (µg/mL)	1.75 (1.19)	2.18 (1.92)
Leucocytes (x10^9^ L)	6.76 (2.29)	7.97 (2.27)
Platelets (x10^9^ L)	264 (67)	252 (79)
Haemoglobin (g/dL)	13.4 (1.2)	14.4 (1.2)
Haematocrit (%)	41.0 (3.2)	43.5 (3.3)
Ferritin (ng/mL)	183 (290)	129 (100)
Albumin (g/dL)	4.6 (0.3)	4.7 (0.7)

Abbreviations: MBI, mindfulness-based intervention; SMT, standard medical therapy

**Table 2:** Between-group comparisons at follow-up (6 months).

VARIABLES	GROUP				MBI minus SMT Follow-up (6 months)		
	Baseline		Follow-up (6 months)				
	MBI	SMT	MBI	SMT	Difference (95% CI)	*P*	ηp^2^
Faecal calprotectin (µg/g)	198 ± 394	222 ± 242	128 ± 226	495 ± 949	−367 (−705 to −29)	0.03	0.085
C-reactive protein (mg/dL)	1.93 ± 2.47	2.46 ± 3.81	2.43 ± 3.05	5.25 ± 7.73	−2.82 (−5.70 to 0.08)	0.05	0.066
Cortisol in hair (µg/mL)	1.75 ± 1.19	2.18 ± 1.92	1.34 ± 1.07	1.94 ± 2.01	−0.60 (−1.27 to 0.08)	0.25	0.036

Abbreviations: MBI, mindfulness-based intervention; SMT, standard medical therapy.

## Discussion

This study examined the effectiveness of a blended MBI intervention which combined internet and face-to-face sessions, in patients diagnosed with IBD. To the best of our knowledge, this is the first study to analyse the efficacy of MBI among patients with IBD by testing inflammation biomarker concentrations. We found lower levels of CRP and FC inflammation biomarkers in the patients in the MBI group compared to those in the SMT group.

It is well known that the intense bowel inflammation which is characteristic of IBD is accompanied by an acute-phase inflammation biomarker response which is detectable in the blood serum. Only a few inflammation blood or serum markers have been extensively validated in IBD, and fewer still are in routine clinical use. However, CRP is one of the most widely available and regularly used of these markers^5^. CRP has a relatively short half-life of 19 hours, making it a more responsive indicator of acute inflammation than most other acute-phase reactants^5^. Here, our results agree with other work which showed that CRP levels decrease as inflammation reduces^1,5,37^.

Our results also highlight similar findings for FC. The recent introduction of faecal markers as a diagnostic tool for studying IBD allows the simple, fast, reliable, non-invasive, and reproducible evaluation of intestinal inflammation. Among these faecal markers, FC is particularly important because it can be used to control the evolution of inflammatory processes in IBD patients. This protein is mainly contained in the cytoplasm of neutrophils and in the membranes of activated monocytes and macrophages. Gastrointestinal tract inflammatory pathologies cause an increase in mucosal permeability that induces the migration of granulocytes and monocytes to the intestinal lumen. Activation and apoptosis of these cells releases a large amount of calprotectin which is excreted in the stool. FC levels also correlate well with leukocyte excretion and intestinal mucosa permeability. Moreover, FC is resistant to bacterial degradation and is stable in faeces, making it a very useful marker for inflammatory bowel activity in clinical practice^4,6,38–40^.

Our results were similar to those published by Gerbarg *et al*. in which patients with IBD that followed a Breath-Body-Mind Workshop showed lower levels of FC and CRP compared with those in the control group^41^. These authors suggest that bidirectional gut–brain inter-communication with the neural, hormonal, and immune system, among others is likely the mechanism underlying this association^42,43^. Indeed, there is evidence that several factors interact in IBD, including activation of inflammatory-responses and the hypothalamic–pituitary–adrenal axis which stimulates areas of the brain that can change patient behaviour and alter the integrity of the blood–brain barrier. More recent work has also highlighted the function of gut microbiota in IBD as well as patient responses to probiotics. In contrast, the role of psychological stress in IBD remains controversial, even though stress and/or traumatic early-life events are known risk factors for the development of this disease, and stress can exacerbate inflammation and induce disease relapses^23,41–43^. Indeed, other authors have noted that patients with CD or UC experience a higher incidence of emotional disorders than the general population and that depression and anxiety can influence the prognosis and severity of these diseases^44^. Therefore, suitable psychological therapies must be considered as part of the optimal treatment of IBD patients^43^.

In contrast to our expectations, MBI did not significantly affect the cortisol levels found in the hair of patients with IBD at six months. There may be several explanations for this result. The use of hair cortisol levels as a chronic stress biomarker has recently become a topic of global interest^23,27^. There is mounting evidence that the cortisol content of hair reflects the corresponding systemic plasma levels of this hormone over time and so these can be used to estimate both psychological and physiological chronic stress and the extent of hypothalamic pituitary–adrenal axis disorders such as Cushing syndrome and Addison disease^23,27,43,45–47^. However, the validity of the methods used to measure cortisol responses has been questioned^48^, and factors including age, sex, hair-washing frequency, hair treatments, or the use of oral contraceptives can produce misleading results^49^. The efficacy of MBI to affect cortisol responses reported by others was also lower than expected. For example, a meta-analysis by Sanada *et al*. reported that age, the number of MBI sessions, and the overall time spent by patients in MBIs significantly affected these responses^50^. No previous studies have analysed the impact of a blended internet and face-to-face intervention on cortisol responses and so, more work will be required to test the impact that the MBI delivery system may have on this biomarker.

Although we found no differences in cortisol between IBD patients in the MBI and the SMT groups at the molecular level, other groups have described relatively high levels of stress and psychological dysfunction among patients with CD or UC^47,51^. Stress increases gastrointestinal (GI) tract permeability and modifies gut microbiota to promote the pathophysiology of IBD through corticotropin-releasing factor (CRF) and urocortin;^47,52–54^ these neuromediators then act on G-protein coupled CRF1 and CFR2 receptors in the brain and GI tract^53^. CRF2 receptors are involved in the perturbation of intestinal permeability. Mastocytes in the mucosa release CRF and urocortin which binds to CRF1 and CRF2 in the same lamina propria, causing these mastocytes to release cytokines and other pro-inflammatory mediators^54^. CRF also increases intestinal permeability by causing mast cells to release tumour necrosis factor alpha and proteases^55^. Thus, targeting CRF receptors with selective antagonists to inhibit mast cell activation is a therapeutic option for chronic inflammatory disorders that are exacerbated by stress.

Chronic early-life stress alters intestinal permeability in rats, which may later sensitise adult rats to visceral hypersensitivity and induce dysbiosis^56^. Stress also inhibits the vagus nerve (VN) and stimulates the sympathetic nervous system via autonomic nervous system-related paraventricular hypothalamic nucleus projection neurons; these are connected to the VN dorsal motor nucleus and sympathetic pre-ganglionic neurons of the spinal cord^53^. Indeed, because the VN exerts an anti-inflammatory effect through its afferent and efferent fibres, stress can be considered a pro-inflammatory process. Thus, an acute stressor in itself can induce a protracted upsurge in pro-inflammatory cytokines, even when the person is no longer exposed to the stressor^57^, all of which can neutralise the critical parasympathetic rebound recovery period. Furthermore, the recovery of parasympathetic-responsiveness can be neutralised by exposure to repeated and/or multiple stressors, in so favouring an allostatic load that dampens the regulatory anti-inflammatory effects of the VN. Hence, stress may tip the balance of the mainly protective effect the VN has on the GI tract epithelial barrier, thus encouraging dysbiosis by disrupting epithelial homeostasis^45,53^.

It is important to mention that this present study was limited by the fact that we did not record patient reported outcomes for IBD (i.e. stress, depression, anxiety, or other QoL measures). Therefore, future research should investigate possible relationships between these outcomes and inflammatory biomarkers. Nonetheless, taking our findings together, MBI likely represents a useful tool that can be incorporated into interventions designed to improve the physiological symptoms of inflammation in patients diagnosed with IBD. In particular, our results indicate that MBI may positively influence (with moderate to large effect sizes) inflammation markers for CRP and FC. Thus, future work should be designed to try to verify these findings by evaluating several physiological indices and to increase our knowledge of the biological systems which benefit from MBI-mediated reductions in stress which can therefore serve as useful biological indicators in these patients.

## Data Availability

Our data are available on request.
